# Chiroptics of In-Plane Excitons

**DOI:** 10.1021/acs.jpca.6c01820

**Published:** 2026-05-31

**Authors:** Sophia Sburlati, Afton Gustafson, Bart Kahr

**Affiliations:** Department of Chemistry, 5894 New York University, 29 Washington Place, New York, New York 10003, United States

## Abstract

The exciton chirality method relates chiroptical activity to absolute stereochemistry, but because the procedure focuses on the geometry of coupled transition electric dipole moments only, the essential roles of magnetic dipole and electric quadrupole transition moments are nonobvious in many applications. These moments become apparent when analyzing in-plane, achiral coupled excitons. In silico C_18_H_2_ polyyne dimers spanning geometries between optically inactive H- and J-aggregate extrema are used to investigate the multipolar response of coupled excited states through the gyration and rotatory strength tensors. As anticipated, the chiroptical response is dominated by the exciton states. The responses are largest in some directions for intermediate geometries and can be reconciled simply with the transition moments derived from structure. The connection between the in-plane moments and the exciton chirality method is established by a minimal symmetry-breaking perturbation that generates circular dichroism couplets while preserving the electronic and pedagogic relationship to the planar systems.

## Graphical abstract

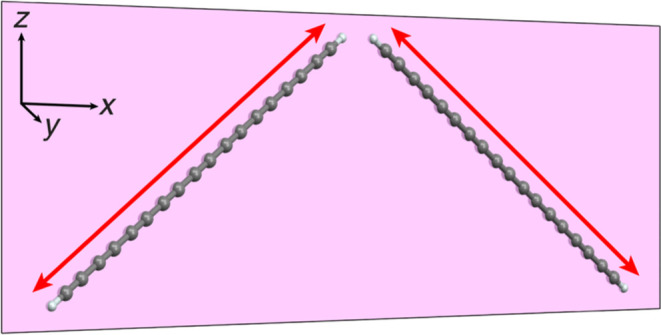

## Introduction

The exciton chirality method (ECM) relates the chiroptical response of coupled electric dipole oscillators to their spatial arrangement and has been widely applied in determinations of absolute configuration.^1–6^ In-plane coupling, however, does not preclude a chiroptical response when optical activity is considered beyond the spatial average. Planar assemblies can support delocalized excited states^7–11^ while also exhibiting anisotropic chiroptical responses suitably oriented. It has been demonstrated that exciton coupling can generate orientation-dependent circular dichroism (CD) in select nonenantiomorphous point groups; achiral systems may feature optical activity via in-plane excitonic interactions.^12–14^

Previously, we investigated computationally the chiroptical consequences of distorting linear C_18_H_2_ (*D*_∞*h*_ symmetry).^15^ Progressive bending and twisting of the polycarbyne chain revealed that bending into *C*_2*v*_ symmetric achiral arcs was significantly more generative of anisotropic optical activity than twisting into helices. Although the spatially averaged optical activity of bent polyynes vanishes, their long-wavelength gyration tensors remain substantial for oblique angles of incidence.

Here, we make C_18_H_2_ optically active by enforcing intermolecular exciton coupling between molecular pairs, showing that the coplanar interactions between two linear C_18_H_2_ molecules provide a pathway to anisotropic optical activity. We examine planar C_18_H_2_ dimers across achiral configurations and analyze their optical activities using the long-wavelength gyration tensor, rotatory strength tensors, and the underlying multipolar moments that give rise to them.

Additionally, planar exciton coupling is explicitly reconnected with the ECM framework by introducing a minimal out-of-plane displacement to a representative chiral dimer geometry, generating a pair of enantiomers. This controlled symmetry-breaking links anisotropic tensorial optical activity and the ECM.

Polyyne chains (carbon wires) are highly reactive, but have been realized through ingenious experiments.^16–19^ Stabilization of linear carbon chains—up to 44 carbon atoms^20^—primarily has been accomplished through the use of bulky end-caps, which suppress cross-linking and decomposition.^21^ Alternative approaches have also been developed, including laser ablation^22^ and encapsulation of polyynes within single-walled carbon nanotubes, the latter of which can stabilize carbon chains extending to thousands of atoms in length.^23^ Specifically, C_18_H_2_ has been prepared by pulsed laser irradiation of toluene.^24^

## Methods

The geometry optimization of C_18_H_2_ and all subsequent computations were performed using Gaussian16 ^25^ at the CAM-B3LYP,^26^ def2-QZVP^27^ levels of theory.^28–33^ The long-wavelength gyration was computed using a linear response within density functional theory at 589 nm. Time-dependent density functional theory was used to calculate absorption spectra, rotatory strengths, and multipolar moments of the first 300 excited states of all structures.

Structures were generated using GaussView.^34^ Dimers were assembled from the optimized C_18_H_2_ molecules pairwise maintaining all atoms in a common plane at nonbonding distances.^35^ Geometries range from parallel (**0**, *D*_2*h*_ symmetry for 0° offset) to increasing oblique angles in increments of 30° (**30**, **60**, **90**, **120**, **150**, *C*_2*v*_ symmetries) to colinear (**180**, *D*_∞*h*_ symmetry). See the bottom row of Figure 1. For **0**, the nonbonding distance between polyynes is 3.4 Å (C···C) and for **30**–**150**, the minimum distance is 2.4 Å (H···H) between polyynes.

**Figure 1:**
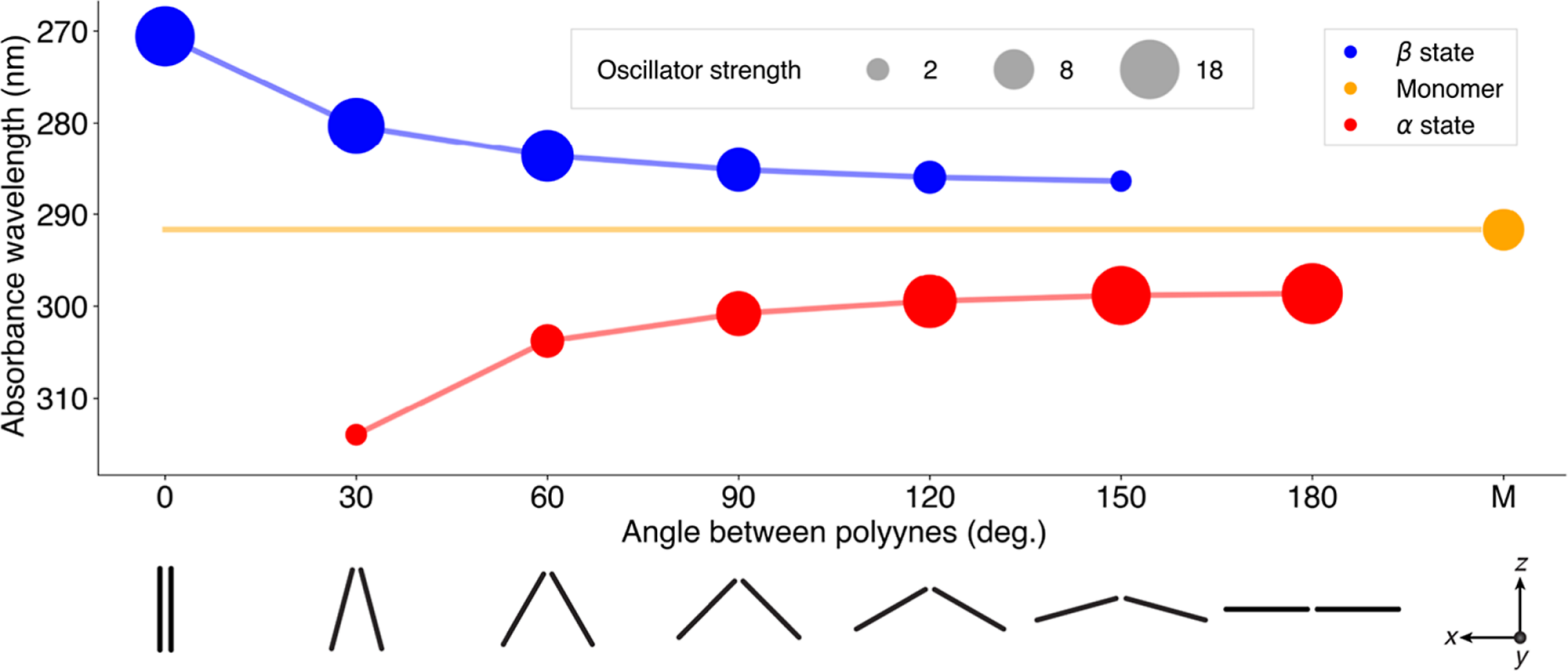
Absorption wavelengths of the exciton-coupled α (red) and β (blue) states for all dimers as a function of interchain angle. The monomer absorption (M, orange) is plotted for reference. Dot areas indicate oscillator strengths. Illustrations of the corresponding dimer geometries are shown along the bottom and oriented with respect to the coordinate system at right.

Exciton coupling arises from interactions between transition electric dipole moments on adjacent chromophores, leading to excited-state delocalization and splitting into two nondegenerate states, α and β, corresponding to antisymmetric and symmetric dipole alignments, respectively. Head-to-tail arrangements form bathochromic J-aggregates, whereas face-to-face arrangements form hypsochromic H-aggregates.^7–10^

Anisotropic CD is quantified by the rotatory strength tensor, while the long-wavelength gyration tensor describes the nonresonant circular birefringence which can be related to specific rotations in solutions. For a given excited state, *n*, the rotatory strength tensor *R* ^(*n*)^ may be constructed from the electric dipole (μ), magnetic dipole (*m*), and electric quadrupole (Θ) transition moments, but is also directly computed by Gaussian16 in the unsymmetrized form. The corresponding gyration tensor element for a transition from state *j* to *n* is obtained from these multipolar moments according to

1 $$g_{\alpha \beta }^{jn}=\frac{1}{2}\left\{\varepsilon_{\alpha \gamma \delta }\left[\frac{1}{3}\omega_{jn}\left(\mu_{\gamma }\Theta_{\delta \beta }-\mu_{\delta }\Theta_{\gamma \beta }\right)-\varepsilon_{\lambda \beta \gamma }\mu_{\delta }m_{\lambda }+\varepsilon_{\lambda \beta \delta }\mu_{\gamma }m_{\lambda }\right]\right\}$$

where ε is the Levi-Civita operator and ω_*jn*_ is the transition energy. Einstein summation over repeated indices is implied. Note that α and β in eqs 1–3 refer to tensor components generally and are not to be confused with the α and β excitonic states.

Elements of the long-wavelength gyration tensor are given by summing over all excited states

2 $$g_{\alpha \beta }=\sum_{j\neq n}\frac{g_{\alpha \beta }^{jn}}{\omega_{jn}^{2}-\omega^{2}}$$

where ω is the nonresonant frequency in the long-wavelength limit. The sum of the rotatory strength tensors over all excited states is proportional to the long-wavelength gyration tensor by

3 $$g_{\alpha \beta }\propto -\sum \left(\frac{1}{\omega_{jn}^{2}-\omega^{2}}\right)R_{\alpha \beta }$$

establishing the formal connection between state-resolved rotatory strength tensors and the frequency-dependent gyration tensor in the long-wavelength limit.^36–41^

## Results and Discussion

### Exciton Coupling

The computed λ_max_ for the C_18_H_2_ monomer is 292 nm. An experimental λ_max_ for C_16_H_2_ was reported as 316 nm.^42^ The absorbance spectrum of the monomer serves as the reference for coupled dimers. The absence of dimer isotropic CD reflects the planar, achiral configurations rather than the absence of excitonically induced optical activity. Absorbance spectra are plotted in Figure S1 and wavelength and oscillator strengths listed in Table S1.

To highlight the geometry-driven exciton coupling across the H to J continuum, the wavelengths and oscillator strengths of α and β are plotted as a function of the angle between component chains in Figure 1. The red and blue dots indicate the α and β states, respectively while the orange dot belongs to the monomer (M) for reference.

Respective transition electric dipole moments (μs) are dictated by exciton symmetry and geometry. The coordinates of μs for the α and β states are listed in Table S2. Figure 2 shows the computed μs plotted as red vectors. Gray arrows drawn along the lengths of the dimer components are the uncoupled moments.

**Figure 2:**
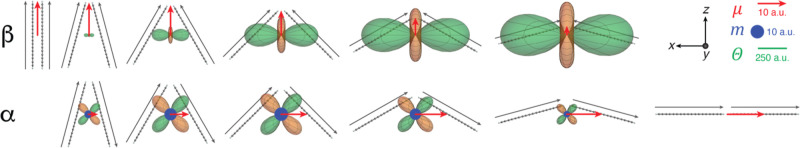
Transition multipole moments of the exciton-coupled states for all dimers. Electric dipole (μ, red arrows), magnetic dipole (*m*, blue circle indicating vectors perpendicular to the plane) and electric quadrupole (symmetric, traceless Θ, green/orange representation surfaces) are shown for α (bottom row) and β (top row) states. Gray arrows illustrate the arrangements of uncoupled transition electric dipoles.

The scalar product of the molecular orbitals that make up α and β states for **90** ^43^ are compared in Figure 3. In-phase regions are yellow, out-of-phase regions are blue, and the gray arrows show the flow of positive charge. The orbital symmetry of α and β transitions confirm a symmetry-consistent excitonic framework for interpreting optical activity.

**Figure 3:**
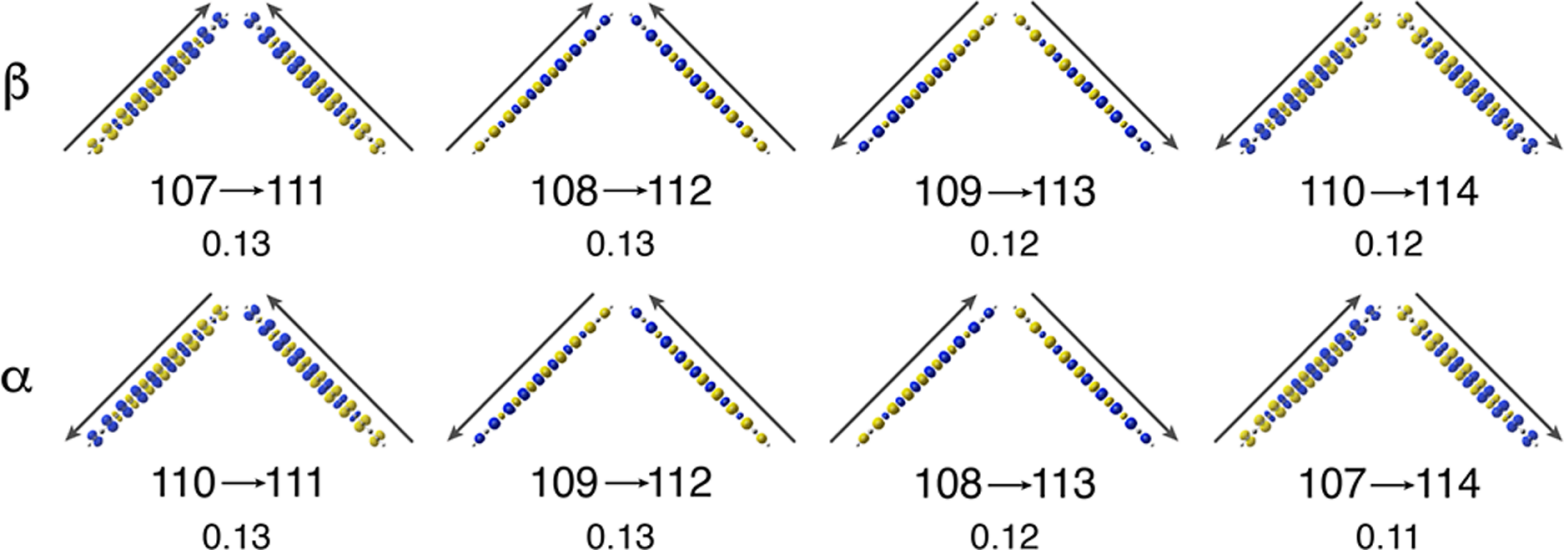
Scalar products of molecular orbitals contributing to the α (bottom row) and β (top row) excitations of **90** (isoval = 0.0004). Yellow denotes in-phase orbital overlap and blue out-of-phase overlap. Gray arrows indicate the direction of charge redistribution during the excitation. Orbital indices identifying each transition are shown below the plots and below that is the square of the pairwise orbital coefficient.

### Anisotropic Optical Activity

M, **0**, and **180** are optically inactive (all tensor elements must be zero) while the *C*_2*v*_ dimers, **30–150**, are optically active when oriented.^44–47^ Two of the diagonalized eigenvalues of *C*_2*v*_-symmetric gyration tensors are equal and opposite (*g*_11_ = −*g*_22_) while the third is zero. The positive nonzero eigenvalues of optically active, planar dimers are 499 bohr^4^ for **30**, 833 for **60**, 978 for **90**, 861 for **120**, and 503 for **150**.

The representation surfaces indicate the observable optical responses that spatial averaging would otherwise render zero (Figure 4).^48–51^ Plotted surfaces compare gyration magnitude as a function of geometry which is largest at **90** and decreases as the dimers approach **0** or **180** (Figure 4).

**Figure 4:**
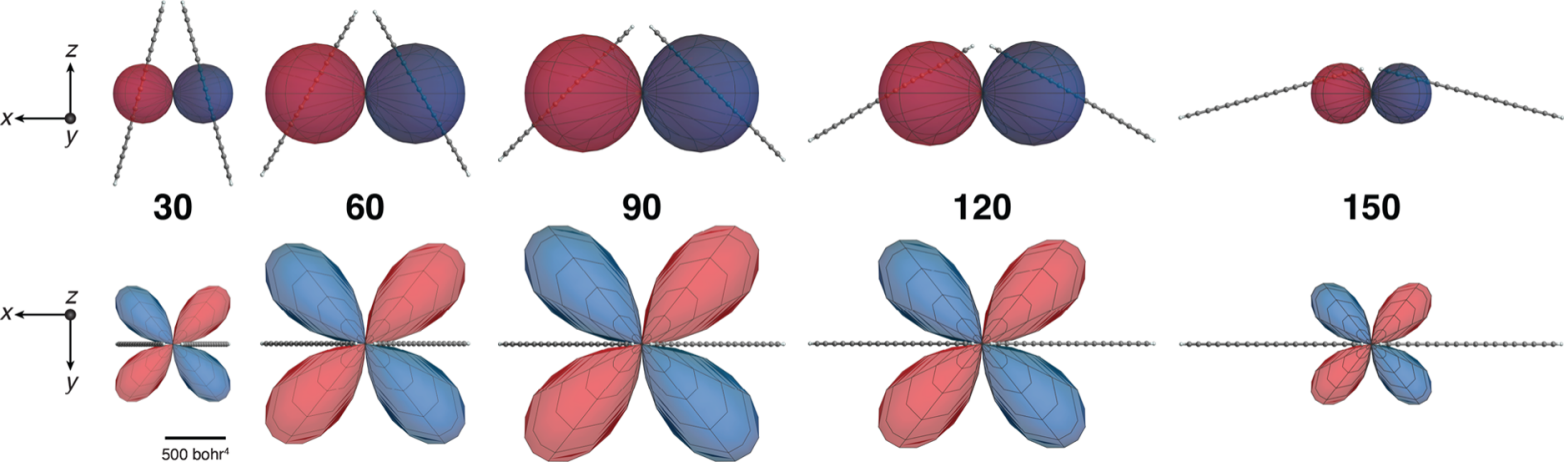
Representation surfaces of the long-wavelength gyration tensors for all optically active, planar dimers. Surface magnitude reflects the anisotropic optical response associated with each geometry. The coordinate systems at left indicate the orientations in each row. Surface colors indicate the sign (red = positive). Gyration as computed is minus the optical rotation.

The gyration of Figure 4 is nonresonant, capturing the overall optical activity of the dimers, but it does not narrate the response. The long-wavelength gyration tensor obtained from linear response theory is formally equivalent to the sum of the state-specific gyration contributions over all excited states. The gyration of a single state is the direct result of the induced multipolar moments of that singular excitation. Therefore, before multipolar moments can be meaningfully analyzed, a state-resolved decomposition of the gyration tensor is useful in the identification of the microscopic source of the observed geometry dependence. The per-state gyration tensors of 300 excited states were calculated from the multipolar moments for all optically active structures. Of the 300 states considered, the gyrotropy is dominated by two state-specific gyration tensors corresponding to the exciton-coupled α and β states. As the sum-over-excited states often requires hundreds of states to achieve convergence to the long wavelength value for small molecules, it can be instructive to identify those systems for which the overall response is reconciled by only a handful of states.^45,52–56^ For all dimers, the sum of the α and β state gyration tensors (α + β) closely reproduces the overall gyration tensor (Table S4). The percent difference between an eigenvalue of the α + β gyration and that of the overall gyration is 23% for **30**, 6% for **60**, 3% for **90**, 3% for **120**, and 2% for **150**. Generally, the linear response theory can be well approximated by just two exciton-coupled states.

Nonzero gyration eigenvalues of the α state, β state, α + β, and overall gyration are plotted according to the left axis of Figure 5 for the planar, optically active dimers. The gyration tensors of the α states and the overall gyration tensors have like-signed directions whereas all β state tensors are opposite of these. The responses largely cancel as expected. The α state eigenvalues are plotted as positive quantities (the same as the overall) and the β state eigenvalues are plotted as negative quantities. The α + β states gyration tensors are also the same sign as the overall gyrations; therefore, the eigenvalues of the sums are also plotted as a positive quantity in dark green at the left of Figure 5. In this format, the oppositely signed but comparably large anisotropic responses of the α and β states are made clear, reflecting distinct, underlying multipolar contributions by the two exciton states. This reflects the oppositely signed components in the CD spectra of systems suited to the ECM.

**Figure 5:**
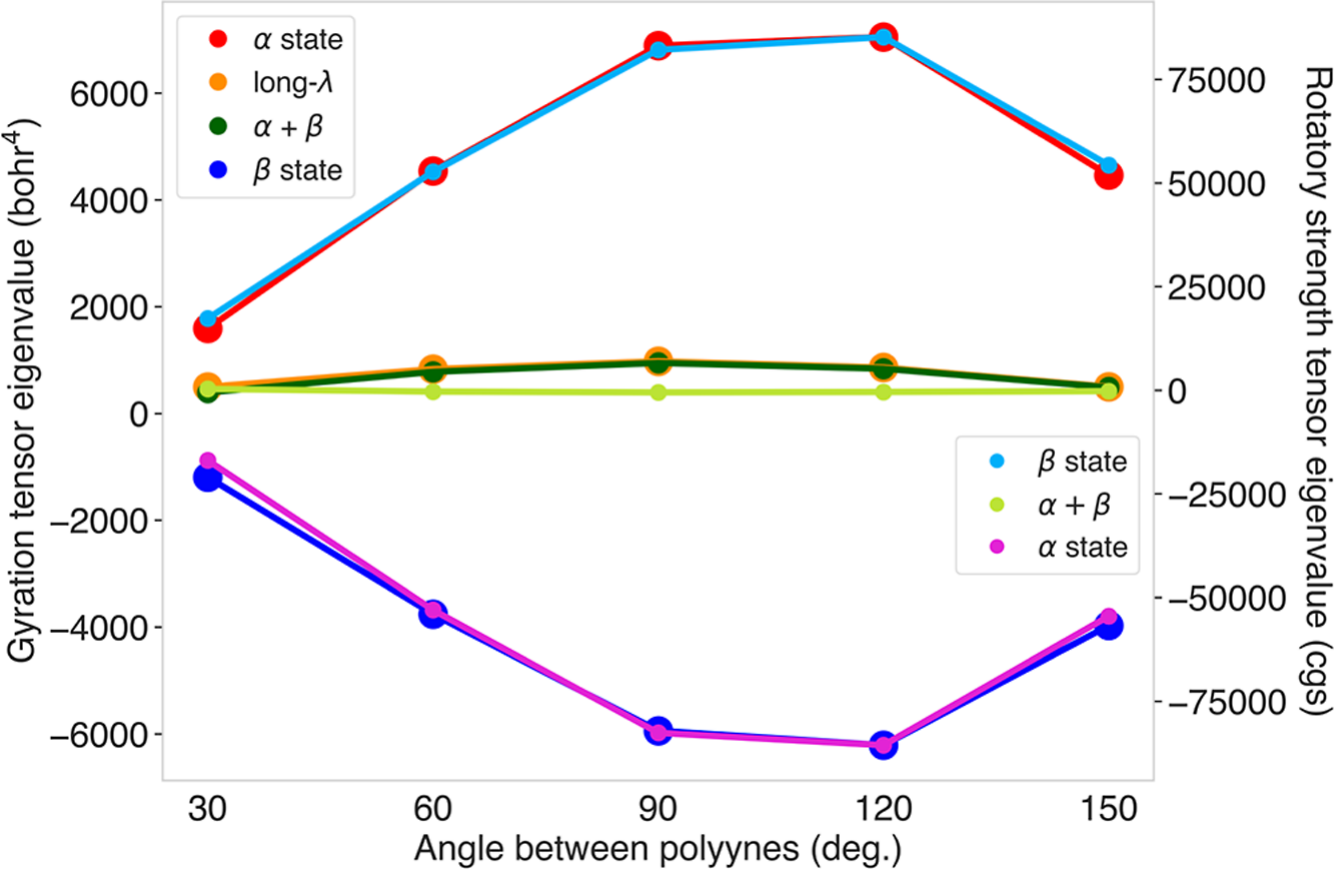
Nonzero eigenvalues of gyration tensors (left axis) and rotatory strength tensors (right axis) for all optically active, planar dimers. Gyration eigenvalues of the α state (red), β state (dark blue), α + β (dark green), and long-wavelength (long-λ, orange) are represented by the top-left legend. Rotatory strength eigenvalues of the α state (magenta), β state (light blue), α + β (light green) are represented by the lower-right legend.

The right–hand axis of Figure 5 pertains to nonzero symmetrized rotatory strength tensor eigenvalues of the α state (magenta), β state (light blue), and α + β state tensors (light green) similarly plotted for optically active, planar dimers (Table S5).

Polarizability tensors with μ coupled to *m* and μ coupled to Θ both contribute to the observed anisotropic response.^37^ The *B*_1_ α state of *C*_2*v*_ dimers have all three moments whereas the *A*_1_ β state have only μ and Θ (see Table S3 for full, traceless Θ tensors). Figure 6 contains the normalized moment magnitudes of the exciton states with α on the left and β on the right, as well as normalized magnitudes of relevant geometric parameters for all optically active planar dimers.

**Figure 6:**
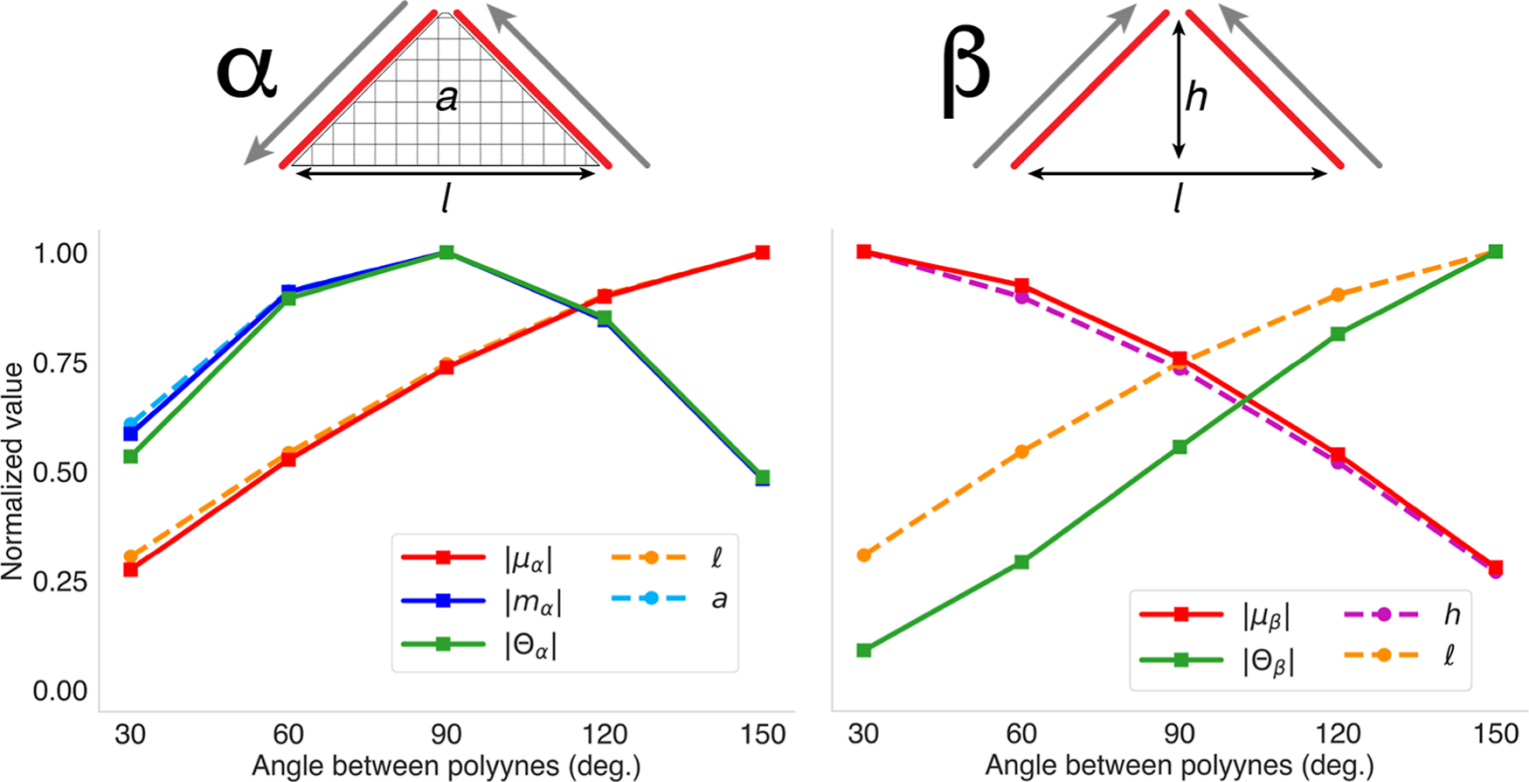
Normalized magnitudes of the transition multipolar moments for the α (left) and β (right) exciton states of optically active, planar dimers. Electric dipole |μ|, magnetic dipole |*m*|, and electric quadrupole |Θ| moment magnitudes are compared with normalized geometric parameters of the dimers. The geometric quantities illustrated above the plots correspond to the triangular area (*a*), base length (*l*), and height (*h*) defined by the dimer geometry.

There is a correspondence between the normalized moment magnitudes and geometric parameters shown in Figure 6. For the α state, |μ| follows the far distance between polyyne ends, denoted as *l* for base length. As dimer angle increases, so too does *l* which supports an increasing vector sum of the proposed antisymmetric uncoupled moments. The α state’s |*m*| and |Θ| both correlate strongly to the triangular areas inscribed by the dimer components (*a*). The connection between *a* and |*m*| follows from a classical description of circulating charge.^57^ The traceless α state Θs are plotted as representation surfaces in the bottom row of Figure 2. They represent square quadrupoles in the plane of the dimer. It follows that the intermediate dimers which maximize *a* would be most supportive of a moment quantifying the square displacement of electric charge.

On the right of Figure 6 are the normalized values of |μ| and |Θ| of the *A*_1_ β state. |μ| correlates to the height of the triangle inscribed by the dimers, *h*. An increase in dimer angle decreases *h* and the vector sum of uncoupled, symmetric dipoles in the *z*-direction. Plotted across the top row of Figure 2 are the traceless β state Θs possessing linear quadrupolar character oriented along the *x*-axis that grows as dimer angle increases from left to right. The linear |Θ|s are consequently maximized by dimers where *l* is largest.

The geometry of **90** facilitates the simultaneous support of μ, *m*, and Θ contributions through *l*, *h*, and *a*, manifesting in the largest optical response. Symmetry restrictions compete and cooperate with dimer geometry to produce multipole moments of the largest magnitude for both exciton states. **150** can best support the linear quadrupole of the β state as it is the longest dimer from end to end, but it is also this geometry that makes it the least suited for the α-state quadrupole which is square-like and supported by *a*. **30** can be rationalized vice versa.

Consistent with this picture, decomposition of the gyration into μ–*m* and μ–Θ contributions show that both channels contribute comparably; for the α state of **90**, μ–*m* and μ–Θ eigenvalues (±3561 and ±3327 bohr^4^, respectively) sum to reproduce the total gyration eigenvalues (±6888 bohr^4^), indicating that the magnitudes of the exciton-state moments encapsulate the relative multipolar contributions. This is true for all the optically active, planar structures.

### Chiral Perturbation of **90**

Chains in **90** were displaced by ± 0.5 Å in the bisecting mirror plane and normal to the diad axis to generate enantiomers. The dimers with positive (*p*) and negative (*m*) handedness produce CD spectra with opposite-signed couplets centered on the slightly perturbed α and β states (Figure 7) consistent with predictions of the ECM (see Table S6 for μ and *m* and Table S7 for Θ). The chiral perturbation is also reflected in tensorial observables, illustrated by the rotatory strength tensor representation surfaces of the enantiomers’ α and β states shown in Figure 7 (pink is positive rotatory strength and blue is negative) alongside corresponding CD peaks where a rotated perspective provides a top-down view of the *C*_2_ dimers (Table S9).

**Figure 7:**
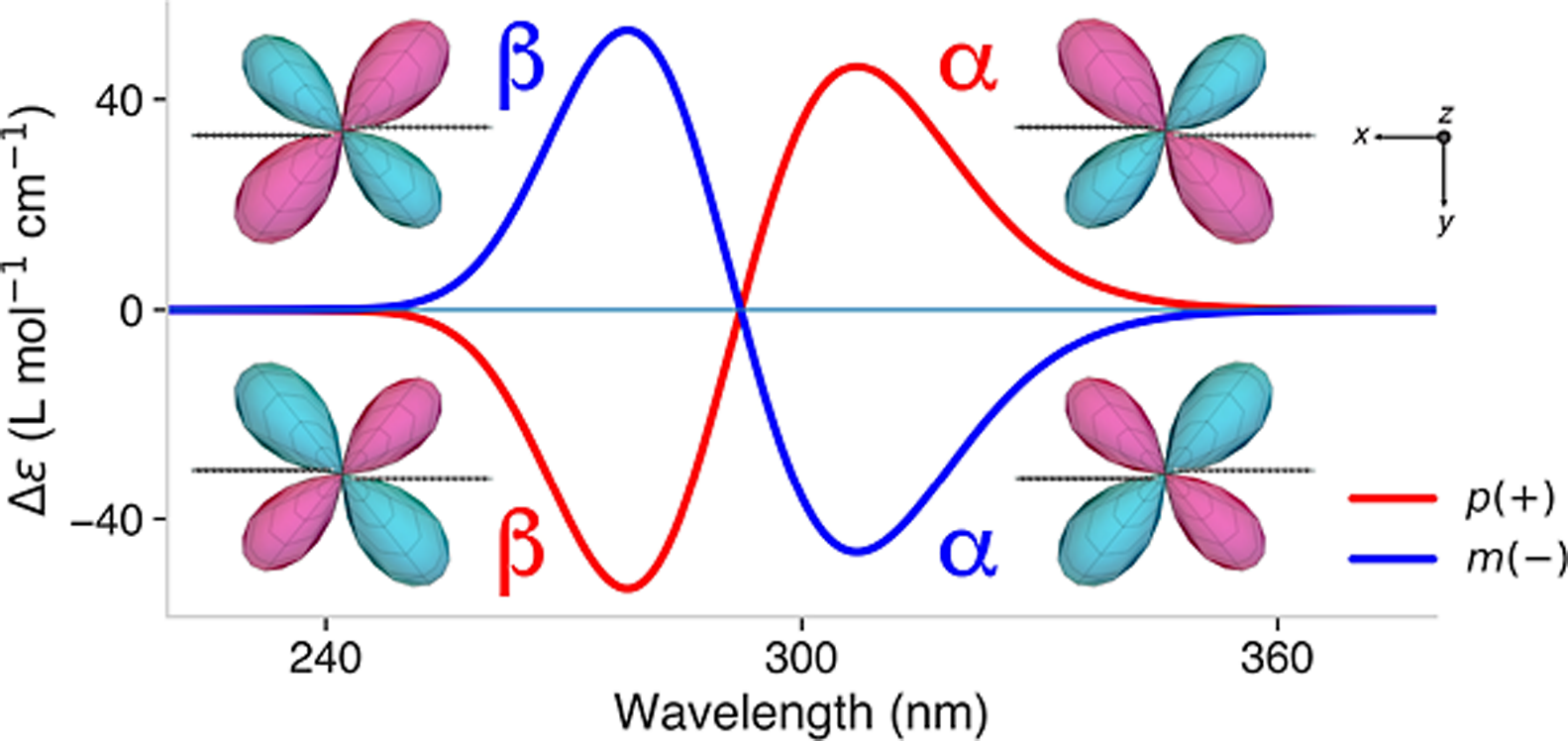
Computed circular dichroism spectra of enantiomeric p (red) and m (blue) dimers generated by a ±0.5 Å displacement of the components of **90**. Rotatory strength tensor representation surfaces for the α and β excitations of each dimer are shown alongside the corresponding spectral feature. The coordinate system used to orient the dimers is indicated at right.

For the achiral systems, the analogy to assigning stereochemistry from the sign of an exciton chirality couplet is to assign the sense of the antisymmetric rotatory strength tensors, that is the absolute sign of the representation surface sampled by the field components of the wave vector.^58^ For a wave vector traveling in the *xy* direction in Figure 7, the field components will align with the positive lobe of the tensor representation surface whose average will likewise be positive as the negative lobe is attenuated.

Like the achiral dimers, the chiral dimers have α and β state gyration tensors that approximately sum to the total long-wavelength tensors. When comparing the largest eigenvalue of the overall gyration tensor and that of the α + β gyration tensor, the enantiomers return a 3% difference (Table S8). These results demonstrate that anisotropic tensorial optical activity in planar exciton-coupled systems represents a symmetry-constrained limit of conventional exciton chirality, becoming manifest as canonical CD couplets upon minimal geometric perturbation.

## Conclusion

A computational study of theoretical dimers composed of two C_18_H_2_ polyynes arranged in the same plane revealed exciton coupling and optical activity. The excitonic states were shown to be the dominant contributors to anisotropic optical activity. Because the α and β excited states are defined by the relative arrangements of uncoupled transition electric dipole moments, the associated multipolar moments were determined by the excitonic symmetry and were therefore qualitatively unique to each state; symmetry defines what is allowed while geometry defines what is expressed. Gyration and rotatory strength bridged exciton coupling in both chiral and achiral systems of oscillators.

While these computations apply to date to theoretical molecular systems, improvements in chiroptical measurements of organized media continue apace.^59,60^ The implications extend to aggregated states of molecular systems, where intermolecular exciton coupling in oriented thin films of achiral chromophores gives rise to in-plane excitonic interactions.^11^ Planar achiral metamaterials can also mimic this type of coupling to confirm the molecular predictions here, as optical activity indistinguishable from that of intrinsically chiral media has been demonstrated in achiral anisotropic planar structures arising from the mutual orientation of the metamaterial and the incident wave.^61–67^ Metal nanostructures provide another promising avenue for experimental research into the optical activity of planar assemblies enhanced by coupling.^68–72^

## Supplementary Material



## Notes

Gaussian16 keywords for all three calculation types Figure S1. Absorbance spectra of planar dimers and monomer Table S1. Absorbance data of planar dimers Table S2. μ and *m* transition moment coordinates of planar dimers Table S3. Θ transition moment tensors of planar dimers Table S4. α, β, α + β gyration eigenvalues of achiral, optically active dimers Table S5. α, β, α + β rotatory strength eigenvalues of achiral, optically active dimers Table S6. μ and *m* transition moment coordinates of chiral dimers Table S7. Θ transition moment tensors of chiral dimers Table S8. Long-wavelength, α, β, α + β gyration eigenvalues of chiral dimers Table S9. α, β, α + β rotatory strength eigenvalues of chiral dimers (PDF)
