# Supplementary material for: Chiroptics of In-Plane Excitons
Source: J Phys Chem A. 2026 May 31;130(23):4378–85. doi: 10.1021/acs.jpca.6c01820 (PMC13267073; doi:10.1021/acs.jpca.6c01820)
Supplement: Supplementary file 1 [file jp6c01820_si_001.pdf]

Chiroptics of In-Plane Excitons

*Sophia Sburlati, Afton Gustafson, Bart Kahr**

Department of Chemistry, New York University, 29 Washington Place, New York, New York, 10003, United States

ASSOCIATED CONTENT

Gaussian16 keywords for all three calculation types

Figure S1. Absorbance spectra of planar dimers and monomer

Table S1. Absorbance data of planar dimers

Table S2. μ and *m* transition moment coordinates of planar dimers

Table S3. *Θ* transition moment tensors of planar dimers

Table S4. α, β, α + β gyration eigenvalues of achiral, optically active dimers

Table S5. α, β, α + β rotatory strength eigenvalues of achiral, optically active dimers

Table S6. μ and *m* transition moment coordinates of chiral dimers

Table S7. *Θ* transition moment tensors of chiral dimers

Table S8. Long-wavelength, α, β, α + β gyration eigenvalues of chiral active dimers

Table S9. α, β, α + β rotatory strength eigenvalues of chiral active dimers

Gaussian16 keywords for all three calculation types

Gaussian16 keywords used to optimize:

#p opt cam-b3lyp/def2qzvp

Gaussian16 keywords used to compute gyration:

#p cam-b3lyp def2qzvp nosymm polar=optrot cphf(rdfreq) iop(10/46=7)

…

589nm

Gaussian16 keywords used to compute 300 excited states:

#p cam-b3lyp def2qzvp nosymm td (full singlets nstates=300, sos) iop(9/33=1)

…

589nm

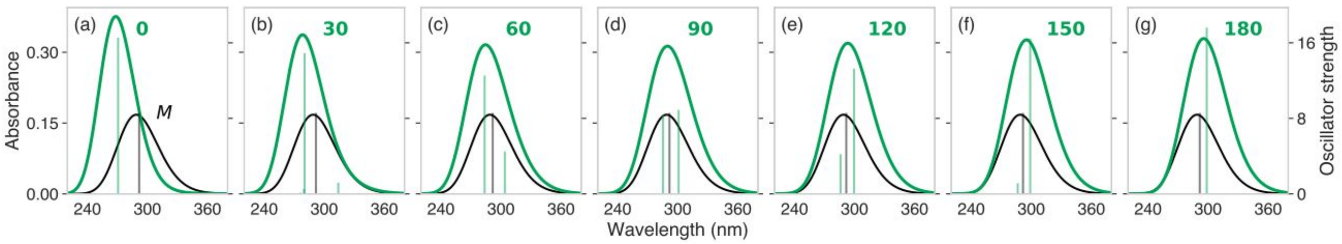

Figure S1. Computed absorbance spectra of dimers in green and monomer in black. S1a., **0**, and S1g., **180**, have only one dimer excitation each as they model ideal H- and J-aggregates, respectively. S1b.-S1f., **30-150**, have both α and β peaks. All spectra have same monomer, *M*, peak plotted for reference.

Table S1. Computed absorbance wavelengths (λ) and oscillator strengths (f) of α and β states of monomer and all planar dimers.

Symmetry	Angle (deg.)	λ_α (nm)	f_α	λ_β (nm)	f_β
$D_{\infty h}$ (monomer)	-	292.7	8.6	-	-
D_{2h}	0	-	-	270.6	16.5
C_{2v}	30	314.0	1.2	280.4	14.9
	60	303.8	4.5	283.6	12.5
	90	300.8	8.9	285.2	8.4
	120	299.5	13.3	286.0	4.2
	150	298.8	16.4	286.4	1.1
$D_{\infty h}$	180	298.6	17.6	-	-

Table S2. Computed velocity gauge electric dipole (μ) and magnetic dipole (m) transition moment coordinates of α and β states of monomer and all planar dimers. All reported μ are divided by the energy of the associated electronic excitation in Hartrees, all m are multiplied by 1/2, and all moments are in a.u.

		μ_{α}			m_{α}			μ_{β}			m_{β}		
Symmetry	Angle (deg.)	x	y	z	x	y	z	x	y	z	x	y	z
$D_{\infty h}$ (monomer)	-	0	0	-9.1	0	0	0	-	-	-	-	-	-
D_{2h}	0	-	-	-	-	-	-	0	0	12.1	0	0	0
C_{2v}	30	-3.5	0	0	0	-7.5	0	0	0	11.7	0	0	0
	60	-6.7	0	0	0	-11.7	0	0	0	10.8	0	0	0
	90	-9.4	0	0	0	-12.9	0	0	0	8.9	0	0	0
	120	-11.4	0	0	0	-10.9	0	0	0	6.3	0	0	0
	150	-12.7	0	0	0	-6.2	0	0	0	3.3	0	0	0
$D_{\infty h}$	180	-13.1	0	0	0	0	0	-	-	-	-	-	-

Table S3. 3x3 computed, traceless, velocity gauge electric quadrupole transition moment (Θ) tensors of α and β states of dimers with non-zero Θ. All reported Θ are multiplied by 3/2, divided by the energy of the associated electronic excitation in Hartrees, and all moments are in a.u.

		Θ_{α}				Θ_{β}		
Angle (deg.)		$\Theta_{x\_}^{\alpha}$	$\Theta_{y\_}^{\alpha}$	$\Theta_{z\_}^{\alpha}$		$\Theta_{x\_}^{\beta}$	$\Theta_{y\_}^{\beta}$	$\Theta_{z\_}^{\beta}$
30	$\Theta_{\_x}^{\alpha}$	0	0	127.3	$\Theta_{\_x}^{\beta}$	45.0	0	0
	$\Theta_{\_y}^{\alpha}$	0	0	0	$\Theta_{\_y}^{\beta}$	0	-32.0	0
	$\Theta_{\_z}^{\alpha}$	127.3	0	0	$\Theta_{\_z}^{\beta}$	0	0	-13.1
		$\Theta_{x\_}^{\alpha}$	$\Theta_{y\_}^{\alpha}$	$\Theta_{z\_}^{\alpha}$		$\Theta_{x\_}^{\beta}$	$\Theta_{y\_}^{\beta}$	$\Theta_{z\_}^{\beta}$
60	$\Theta_{\_x}^{\alpha}$	0	0	-213.2	$\Theta_{\_x}^{\beta}$	166.4	0	0
	$\Theta_{\_y}^{\alpha}$	0	0	0	$\Theta_{\_y}^{\beta}$	0	-91.0	0
	$\Theta_{\_z}^{\alpha}$	-213.2	0	0	$\Theta_{\_z}^{\beta}$	0	0	-75.5

		$\Theta_{x\_}{}^{\alpha}$	$\Theta_{y\_}{}^{\alpha}$	$\Theta_{z\_}{}^{\alpha}$		$\Theta_{x\_}{}^{\beta}$	$\Theta_{y\_}{}^{\beta}$	$\Theta_{z\_}{}^{\beta}$
90	$\Theta_{\_x}{}^{\alpha}$	0	0	-238.3	$\Theta_{\_x}{}^{\beta}$	324.7	0	0
	$\Theta_{\_y}{}^{\alpha}$	0	0	0	$\Theta_{\_y}{}^{\beta}$	0	-167.2	0
	$\Theta_{\_z}{}^{\alpha}$	-238.3	0	0	$\Theta_{\_z}{}^{\beta}$	0	0	-157.6
		$\Theta_{x\_}{}^{\alpha}$	$\Theta_{y\_}{}^{\alpha}$	$\Theta_{z\_}{}^{\alpha}$		$\Theta_{x\_}{}^{\beta}$	$\Theta_{y\_}{}^{\beta}$	$\Theta_{z\_}{}^{\beta}$
120	$\Theta_{\_x}{}^{\alpha}$	0	0	202.9	$\Theta_{\_x}{}^{\beta}$	-480.0	0	0
	$\Theta_{\_y}{}^{\alpha}$	0	0	0	$\Theta_{\_y}{}^{\beta}$	0	242.3	0
	$\Theta_{\_z}{}^{\alpha}$	202.9	0	0	$\Theta_{\_z}{}^{\beta}$	0	0	237.8
		$\Theta_{x\_}{}^{\alpha}$	$\Theta_{y\_}{}^{\alpha}$	$\Theta_{z\_}{}^{\alpha}$		$\Theta_{x\_}{}^{\beta}$	$\Theta_{y\_}{}^{\beta}$	$\Theta_{z\_}{}^{\beta}$
150	$\Theta_{\_x}{}^{\alpha}$	0	0	116.2	$\Theta_{\_x}{}^{\beta}$	-592.6	0	0
	$\Theta_{\_y}{}^{\alpha}$	0	0	0	$\Theta_{\_y}{}^{\beta}$	0	296.9	0
	$\Theta_{\_z}{}^{\alpha}$	116.2	0	0	$\Theta_{\_z}{}^{\beta}$	0	0	295.7

Table S4. Gyration tensor eigenvalues (g) calculated from computed multipolar moments of α and β states for optically active, planar dimers. $g^{\alpha}+g^{\beta}$ eigenvalues generated by adding g^{α} and g^{β} then diagonalizing. All eigenvalues are represented in units of $bohr^4$.

	g^{α}			g^{β}			$g^{\alpha}+g^{\beta}$		
Angle (deg.)	$g_{11}{}^{\alpha}$	$g_{22}{}^{\alpha}$	$g_{33}{}^{\alpha}$	$g_{11}{}^{\beta}$	$g_{22}{}^{\beta}$	$g_{33}{}^{\beta}$	$g_{11}{}^{\alpha+\beta}$	$g_{22}{}^{\alpha+\beta}$	$g_{33}{}^{\alpha+\beta}$
30	-1591	1591	0	1196	-1196	0	-394	394	0
60	-4540	4540	0	3759	-3759	0	-781	781	0
90	-6888	6888	0	5940	-5940	0	-948	948	0
120	-7051	7051	0	6211	-6211	0	-840	840	0
150	-4463	4463	0	3973	-3973	0	-491	491	0

Table S5. Total rotatory strength tensor eigenvalues (rs) symmetrized from computation output files of α and β states for optically active, planar dimers. rs^{α}+rs^{β} eigenvalues generated by adding rs^{α} and rs^{β} then diagonalizing. All eigenvalues are represented in units of cgs.

	rs^{α}			rs^{β}			$rs^{\alpha}+rs^{\beta}$		
Angle (deg.)	rs_{11}^{α}	rs_{22}^{α}	rs_{33}^{α}	rs_{11}^{β}	rs_{22}^{β}	rs_{33}^{β}	$rs_{11}^{\alpha+\beta}$	$rs_{22}^{\alpha+\beta}$	$rs_{33}^{\alpha+\beta}$
30	-16957	16957	0	17283	-17283	0	327	-327	0
60	-53014	53014	0	52708	-52708	0	306	-306	0
90	-82616	82616	0	82115	-82115	0	501	-501	0
120	-85594	85594	0	85206	-85206	0	388	-388	0
150	-54500	54500	0	54300	-54300	0	200	-200	0

Table S6. Computed velocity gauge electric dipole (μ) and magnetic dipole (m) transition moment coordinates of α and β states of chiral dimers. All reported μ are divided by the energy of the associated electronic excitation in Hartrees, all m are multiplied by 1/2, and all moments are in a.u.

		μ_{α}			m_{α}			μ_{β}			m_{β}		
Symmetry	Enantiomer	x	y	z	x	y	z	x	y	z	x	y	z
C_2	p	-9.4	0.03	0	-0.7	-12.9	0	0	0	8.9	0	0	-0.7
	m	-9.4	-0.03	0	0.7	-12.9	0	0	0	8.9	0	0	0.7

Table S7. 3x3 computed, traceless, velocity gauge electric quadrupole transition moment (Θ) tensors of α and β states of chiral dimers. All reported Θ are multiplied by 3/2, divided by the energy of the associated electronic excitation in Hartrees, and all moments are in a.u.

		Θ_α				Θ_β		
Enantiomer		$\Theta_{x\_}^{\alpha}$	$\Theta_{y\_}^{\alpha}$	$\Theta_{z\_}^{\alpha}$		$\Theta_{x\_}^{\beta}$	$\Theta_{y\_}^{\beta}$	$\Theta_{z\_}^{\beta}$
p	$\Theta_{\_x}^{\alpha}$	0	0	-239.0	$\Theta_{\_x}^{\beta}$	324.9	-12.4	0
	$\Theta_{\_y}^{\alpha}$	0	0	12.7	$\Theta_{\_y}^{\beta}$	-12.4	-167.1	0
	$\Theta_{\_z}^{\alpha}$	-239.0	12.7	0	$\Theta_{\_z}^{\beta}$	0	0	-157.8
		$\Theta_{x\_}^{\alpha}$	$\Theta_{y\_}^{\alpha}$	$\Theta_{z\_}^{\alpha}$		$\Theta_{x\_}^{\beta}$	$\Theta_{y\_}^{\beta}$	$\Theta_{z\_}^{\beta}$
m	$\Theta_{\_x}^{\alpha}$	0	0	-239.0	$\Theta_{\_x}^{\beta}$	324.9	12.4	0
	$\Theta_{\_y}^{\alpha}$	0	0	-12.7	$\Theta_{\_y}^{\beta}$	12.4	-167.1	0
	$\Theta_{\_z}^{\alpha}$	-239.0	-12.7	0	$\Theta_{\_z}^{\beta}$	0	0	-157.8

Table S8. Gyration tensor eigenvalues (g) computed at the long-wavelength and calculated from computed multipolar moments of α and β states for optically active, chiral dimers. $g^{\alpha}+g^{\beta}$ eigenvalues generated by adding g^{α} and g^{β} then diagonalizing. All eigenvalues are represented in units of $bohr^4$.

	g			g^{α}			g^{β}			$g^{\alpha}+g^{\beta}$		
Enantiomer	g_{11}	g_{22}	g_{33}	g_{11}^{α}	g_{22}^{α}	g_{33}^{α}	g_{11}^{β}	g_{22}^{β}	g_{33}^{β}	$g_{11}^{\alpha+\beta}$	$g_{22}^{\alpha+\beta}$	$g_{33}^{\alpha+\beta}$
p	902	-991	-17	6529	-7224	-26	-5652	6262	0	877	-962	-26
m	-902	991	17	-6529	7224	26	5652	-6262	0	-877	962	26

Table S9. Total rotatory strength tensor eigenvalues (rs) symmetrized from computation output files of α and β states for optically active, chiral dimers. All eigenvalues are represented in units of cgs.

	rs^{α}			rs^{β}		
Enantiomer	rs_{11}^{α}	rs_{22}^{α}	rs_{33}^{α}	rs_{11}^{β}	rs_{22}^{β}	rs_{33}^{β}
p	86931	-78570	315	-86464	78042	0
m	-86931	78570	-315	86464	-78042	0
